# Development of a colloidal gold immunochromatographic strip for the simultaneous detection of porcine epidemic diarrhea virus and transmissible gastroenteritis virus

**DOI:** 10.3389/fmicb.2024.1418959

**Published:** 2024-06-19

**Authors:** Jinzhu Zhou, Wei Wu, Dandan Wang, Wei Wang, Xinjian Chang, Yunchuan Li, Jizong Li, Baochao Fan, Junming Zhou, Rongli Guo, Xuejiao Zhu, Bin Li

**Affiliations:** ^1^Institute of Veterinary Medicine, Jiangsu Academy of Agricultural Sciences, Key Laboratory of Veterinary Biological Engineering and Technology Ministry of Agriculture, Jiangsu Key Laboratory for Food Quality and Safety-State Key Laboratory Cultivation Base of Ministry of Science and Technology, Nanjing, China; ^2^Jiangsu Co-Innovation Center for the Prevention and Control of Important Animal Infectious Disease and Zoonoses, Yangzhou University, Yangzhou, China; ^3^Guotai (Taizhou) Center of Technology Innovation for Veterinary Biologicals, Taizhou, China; ^4^Fujian Agricultural and Forestry University, Fuzhou, China; ^5^School of Food and Biological Engineering, Jiangsu University, Zhenjiang, China

**Keywords:** porcine epidemic diarrhea virus, transmissible gastroenteritis virus, colloidal gold immunochromatographic strip assay, monoclonal antibodies, RT-PCR

## Abstract

In recent years, porcine diarrhea-associated viruses have caused significant economic losses globally. These viruses present similar clinical symptoms, such as watery diarrhea, dehydration, and vomiting. Co-infections with porcine epidemic diarrhea virus (PEDV) and transmissible gastroenteritis virus (TGEV) are common. For the rapid and on-site preliminary diagnosis on the pig farms, this study aimed to develop a colloidal gold immunochromatography assay (GICA) strip for the detection of PEDV and TGEV simultaneously. The GICA kit showed that there was no cross-reactivity with the other five common porcine viruses. With visual observation, the lower limits were approximately 104 TCID50/mL and 104 TCID50/mL for PEDV and TGEV, respectively. The GICA strip could be stored at 4°C or 25°C for 12 months without affecting its efficacy. To validate the GICA strip, 121 clinical samples were tested. The positive rates of PEDV and TGEV were 42.9 and 9.9%, respectively, and the co-infection rate of the two viruses was 5.8% based on the duplex GICA strip. Thus, the established GICA strip is a rapid, specific, and stable tool for on-site preliminary diagnosis of PEDV- and TGEV-associated diarrhea.

## 1 Introduction

Porcine epidemic diarrhea virus (PEDV) belongs to the genus *Alphacoronavirus* in the family *Coronaviridae* of the order *Nidovirales* and causes acute diarrhea, vomiting, dehydration, and high mortality in neonatal piglets (Jung et al., 2020). PEDV was identified over 30 years ago (Wood, 1977). However, it was not until 2013 that a new highly virulent PEDV strain appeared in the United States, subsequently causing the disease to emerge worldwide (Stevenson et al., 2013). PEDV has a single-stranded positive-sense RNA genome, which is approximately 28 kb in length and encodes four structural proteins, including spike (S), membrane (M), envelope (E), and nucleocapsid (N) proteins, and 16 non-structural proteins (NSP1–NSP16) (Kocherhans et al., 2001). The N protein is located in the endoplasmic reticulum (ER) (Xu et al., 2013) and can bind viral genomic RNA and interact with other proteins to protect the viral genome (McBride et al., 2014). Furthermore, the N protein is highly conserved and can be used as an accurate diagnostic target for PEDV infection (Song and Park, 2012).

Transmissible gastroenteritis virus (TGEV) also belongs to the genus *Alphacoronavirus* within the subfamily *Coronaviridae*, which are single-stranded, positive-sense RNA viruses (Alonso et al., 2002). All pigs are susceptible to TGEV, which causes watery diarrhea, vomiting, dehydration, and high mortality in piglets less than 2 weeks old (Garwes, 1988). The genome of TGEV is approximately 28.5 kb in length, and the open reading frame (ORF) arrangement is in the order of 5’-ORF1a-ORF1b-ORF2-ORF3-ORF4-ORF5-ORF6-ORF7-3′. ORF2 and ORF4–6 encode four structural proteins, namely spike (S), membrane (M), envelope (E), and nucleocapsid (N) proteins, respectively (Alonso et al., 2002). As with PEDV, the N protein of TGEV is also highly conserved and is the main target for detection (Zhang et al., 2018).

All piglets are susceptible to PEDV and TGEV and can be infected with both viruses at the same time. In most veterinary diagnosis laboratories, currently reported detection methods include virus isolation, immunofluorescence assays, and molecular detection techniques, such as reverse transcription-polymerase chain reaction (RT-PCR) and TaqMan-based real-time quantitative reverse transcription-polymerase chain reaction (RT-qPCR) for PEDV and TGEV detection (Song et al., 2006; Kim et al., 2007). Although these methods have high accuracy and sensitivity, they are often time-consuming and laborious and require specific equipment and well-trained technicians. Furthermore, it is also not suitable for point-of-care testing.

The immunochromatographic assay (ICA) labeled with colloidal gold, which is a technique based on specific antigen–antibody reactions, has proven to be a useful tool because it can be completed within 10 min and does not require specialized equipment or complicated handling procedures for detection (Zhou et al., 2012). In this study, monoclonal antibodies (McAbs) against PEDV and TGEV were prepared. A GICA strip for simultaneously detecting PEDV and TGEV was developed using purified McAbs against PEDV and TGEV N proteins. The method was simple, rapid, and specific for the detection of PEDV and TGEV, and suitable for detecting clinical samples on-site.

## 2 Materials and methods

### 2.1 Viruses

Viruses, including PEDV AH2012 strain (GenBank: KC210145) (Fan et al., 2017), TGEV JS2012 strain (GenBank: KT696544) (Guo et al., 2020), porcine rotavirus (PoRV) NJ2012 strain (GenBank: MT874983-MT874993) (Zhou et al., 2021), porcine deltacoronavirus (PDCoV) CZ2020 strain (GenBank: OK546242) (Li et al., 2022), classical swine fever virus (CSFV) vaccine strain (Shimen/HVRI)(GenBank: AY775178.2), porcine reproductive and respiratory syndrome virus (PRRSV) vaccine strain (JXA1R) (GenBank: KM659203.1) and pseudorabies virus (PRV) Bartha-K61 strain (GenBank:KY398733.1), were preserved in the Key Laboratory of Veterinary Biological Engineering and Technology, Ministry of Agriculture, P.R. China (Nanjing, China).

### 2.2 Production of McAbs against PEDV and TGEV

McAbs against PEDV and TGEV were prepared following a standard procedure (Greenfield, 2012). Six-week-old female BALB/c mice were immunized subcutaneously with a purified nucleocapsid of PEDV and TGEV by *Escherichia coli* electrocompetent cells (strain BL21), as described previously (Chen et al., 2017). Titers of the serum collected from these immunized mice and the screen of hybridomas were determined by indirect enzyme-linked immunosorbent assay (ELISA). The hybridoma strains of selected McAbs were stored in liquid nitrogen until use. For the preparation of ascites, hybridoma cell lines were injected into the abdominal cavity of female mice after pretreatment with liquid paraffin as described previously (Mahana and Paraf, 1993).

### 2.3 Western blot

The purified recombinant N protein of PEDV and TGEV with loading buffer was boiled at 100°C for 5 min. Equal amounts of recombinant protein samples were transferred onto a 0.45-μm PVDF membrane (Millipore, Billerica, MA, United States). After being blocked with Tris-buffered saline containing Tween 20 (TBST) containing 10% (w/v) non-fat milk for 2 h, the membranes were incubated with McAbs-1 and McAbs-2 against PEDV and McAbs-1 and McAbs-2 against TGEV at 4°C overnight, respectively. After being washed three times with TBST, the membranes were incubated with the horseradish peroxidase (HRP)-conjugated goat anti-mouse second antibodies (Beyotime) at 37°C for 1 h. Protein bands were detected using the DAB HRP Color Development Kit (Boster Biological Technology Co., Ltd).

### 2.4 Indirect immunofluorescence assay (IFA)

Briefly, PEDV was inoculated into Vero cells and fixed with methyl alcohol, then blocked with 5% skim milk. Following this, the cells are incubated with McAbs-1 and McAbs-2 against PEDV at 37°C for an hour, and incubated with goat anti-mouse IgG conjugated with FITC (Boster, China) (1:500) for an additional hour. Similarly, TGEV was inoculated into ST cells and fixed with methyl alcohol, then blocked with 5% skim milk. Following this, the cells are incubated with McAbs-1 and McAbs-2 against TGEV at 37°C for an hour, and incubated with goat anti-mouse IgG conjugated with FITC (Boster, China) (1,500) for an additional hour. Finally, the cells were observed under a fluorescence microscope (Olympus IX-51, Japan). Uninfected cells served as a negative control. Furthermore, the nuclei were stained with 4′,6-diamidino-2-phenylindole (DAPI) for 5 min at RT and washed three times. Images were examined using a fluorescence microscope (Olympus IX-15) (Guo et al., 2020).

### 2.5 Preparation of the GICA strip

**Figure 1:**
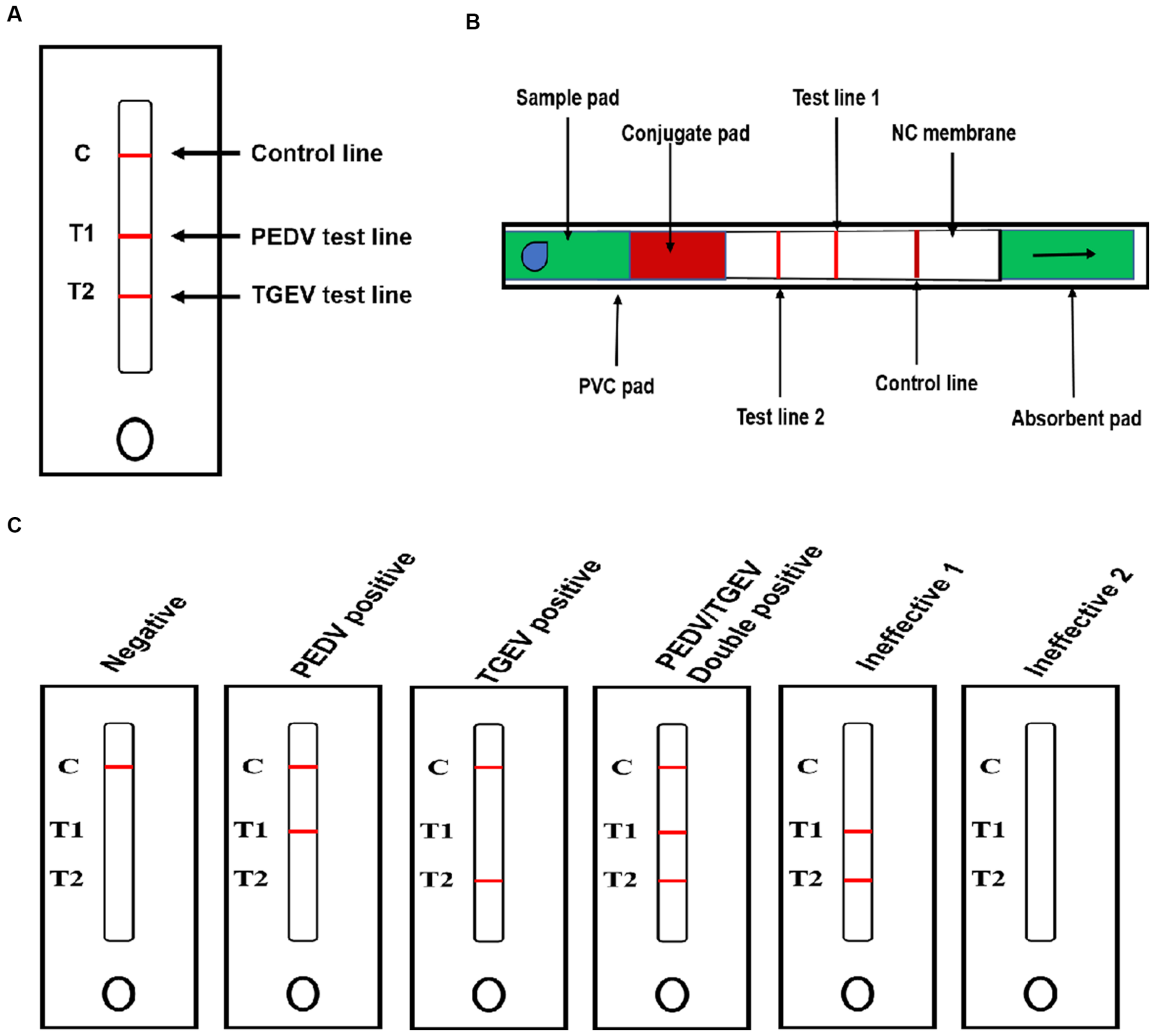
Schematic diagram of the GICA strip. **(A)** External structure of rapid test strip; **(B)** internal structure of rapid test strip; and **(C)** schematic diagram for reading detected results.

The colloidal gold-labeled McAbs were added dropwise to the conjugate pad and dried under a fan for 2 h. The GICA kit includes a sample pad, a conjugate pad, a nitrocellulose membrane, an absorbent pad, and a PVC pad (Yu et al., 2018). The PVC backing card was used as the bottom of the test strip, the nitrocellulose membrane was attached to the center of the PVC backing card, the conjugate pad was attached to the end of the membrane, overlapping by 2 mm, and then the sample pad was attached to the end of the conjugate pad with 1.5 mm overlap. The absorbent pad was attached to the top of the membrane with a 2 mm overlap. Goat anti-mouse IgG antibody, mouse anti-PEDV, and anti-TGEV antibodies were fixed on a nitrocellulose membrane at three discrete zones, with a volume of 1 μL/cm^2^ to form control line (C line), PEDV test line (T1 line), and TGEV test line (T2 line) as Figure 1A, respectively, with Biodot equipment (BioDot, Inc., ZX1000, United States). A schematic diagram of the test strip is shown in Figure 1B. These strips were stored in a desiccator at 4°C prior to use.

### 2.6 The procedure and principle of the GICA strip

Stools and intestinal contents samples (diluted 1:10 in PBS) were thoroughly homogenized using a tissue fragmentation apparatus. The homogenates were centrifuged at 10,000 × g for 5 min, and the supernatants were used for antigen detection. A 100-μL processed supernatant was transferred into the sample well (S-well). Because of the color reaction of GICA, the results could be checked for 5–10 min with the naked eye without any special equipment. The principle of a GICA kit is presented in Figure 1C. If the sample contained PEDV or TGEV, the detected virus formed a complex with the gold-labeled McAbs. In the presence of the target virus, the test line showed color or weak color, while the control line showed color, indicating that the test results were positive or weakly positive. If the samples did not contain PEDV or TGEV, the control line showed color while the test lines were absent, indicating that the test results were negative. The absence of the control line indicated that the IC strip was ineffective. The presence or absence of the control line was used as the standard to evaluate whether the prepared strip was valid (color/present) or invalid (colorless/absent).

### 2.7 Specificity evaluation of the GICA strip

To evaluate the specificity of the colloidal gold test strip, PDCoV, PoRV, PRV, CSFV, and PRRSV were simultaneously detected. Containing 10^5^ TCID_50_, different viruses were added to the sample well of the GICA kit and incubated for 10 min at room temperature, respectively. The results were recorded using photographs.

### 2.8 Sensitivity evaluation of the GICA strip

The PEDV/AH2012 strain and TGEV/JS2012 strain were propagated in Vero cells and ST cells, respectively. The TCID_50_ of viruses was calculated using the Reed–Muench method. The viruses were diluted to 1.0 × 10^6^, 1.0 × 10^5^, 1.0 × 10^4^, and 1.0 × 10^3^ TCID_50_/mL of cultured PEDV and TGEV, then were used to determine the sensitivity of the rapid detection strip. The diluted viruses were added to the sample well of the test strip and incubated for 10 min at room temperature, respectively. Finally, the results were recorded using photographs.

### 2.9 Stability evaluation of the GICA strip

The strips were tested to determine their specificity in detecting PEDV/AH2012 and TGEV/JS2012 viruses following storage at 4°C or 25°C for 12 months. A total of 100 μL of PEDV/AH2012 and TGEV/JS2012 (1.0 × 10^6^ TCID_50_/mL) virus samples were added to the sample well, and PBS was used as a negative control. After incubating for 10 min at room temperature, the results were recorded using photographs.

### 2.10 Detection of clinical samples

A total of 121 rectal swab samples, including feces and intestinal contents, were collected from pig farms in three provinces in China between January 2018 and December 2019 (Table 1). All samples were tested using RT-PCR with the primers (Table 2) and the GICA kit at the same time.

**Table 1:** Information on clinical field samples from swine farms.

No. of pig farm	The position of pig farms	Amount of samples collection	Types of sample
1	Nanjing, Jiangsu,	10 (1–10)	Fecal swabs
2	Xuzhou, Jiangsu	14 (11–24)	Fecal swabs
3	Wuxi, Jiangsu	12 (25–36)	Fecal swabs
4	Huaian, Jiangsu	8 (37–44)	Fecal swabs
5	Chuzhou, Anhui	18 (45–62)	Fecal swabs
6	Bengbu, Anhui	10 (63–72)	Fecal swabs
7	Ma’anshan, Anhui	6 (73–78)	Fecal swabs
8	Bozhou, Anhui	9 (79–87)	Fecal swabs
9	Wenzhou, Zhejiang	10 (88–97)	Fecal swabs
10	Hangzhou, Zhejiang,	16 (98–113)	Fecal swabs
11	Xiaoshan, Zhejiang	8 (114–121)	Fecal swabs

**Table 2:** Primers used for RT-PCR assays.

Primers	Target gene	Product size (nt)	Sequence (5′-3′)
PEDV-F	Nucleoprotein (N)	796	GCATTTCTACTACCTCGGAA
PEDV-R			GCGATCTGAGCATAGCCTGA
TGEV-F	Membrane (M)	544	GCGTCTGATTGTGAGTCATG
TGEV-R			AATAGTCCTGCTGGGTAATG

### 2.11 Ethics statement

All applicable international, national, and/or institutional guidelines for the care and use of animals were followed by the Jiangsu Academy of Agricultural Sciences Experimental Animal Ethics Committee (NKYVET 2015–0127).

## 3 Results

### 3.1 Characterization of anti-PEDV and anti-TGEV McAbs

**Figure 2:**
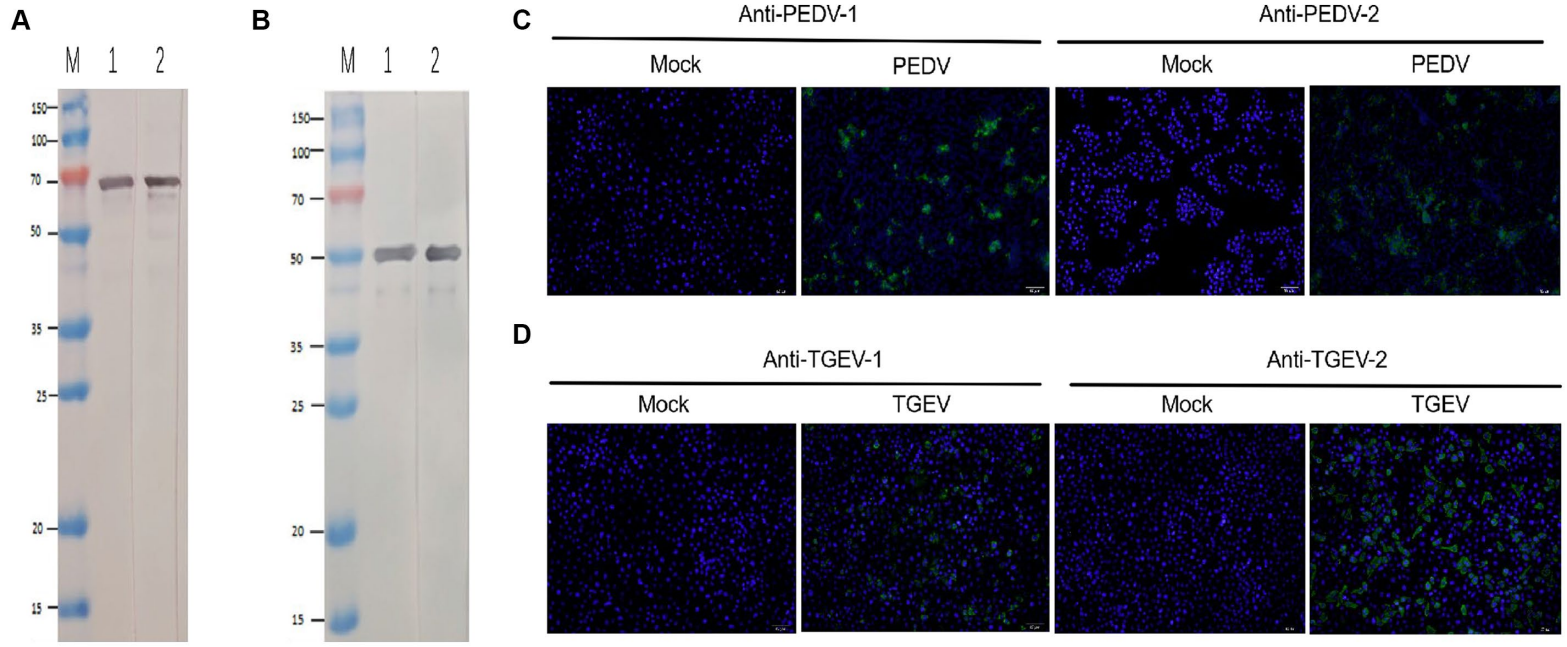
Identification of PEDV and TGEV monoclonal antibody. **(A)** The PEDV N protein was run for Western blotting using anti-PEDV McAb-1 and anti-PEDV McAb-2. **(B)** The TGEV N protein was run for Western blotting using anti-TGEV McAb-1 and anti-TGEV McAb-2. **(C)** IFA was stained for PEDV (green) and nuclei (DAPI, blue) with anti-PEDV McAb-1 and anti-PEDV McAb-2. **(D)** IFA was stained for PEDV (green) and nuclei (DAPI, blue) with anti-TGEV McAb-1 and anti-TGEV McAb-2.

Positive hybridoma cells against PEDV and TGEV were detected, designated as anti-PEDV McAb-1, anti-PEDV McAb-2, anti-TGEV McAb-1, and anti-TGEV McAb-2 by indirect ELISA and cloned by limiting dilution. The specificity of generated McAbs to PEDV and TGEV was determined using Western blot and IFA. Western blot results showed that the two anti-PEDV McAbs specifically reacted with the PEDV N protein via SDS-PAGE (Figure 2A), and the two anti-TGEV McAbs specifically reacted with the TGEV N protein via SDS-PAGE (Figure 2B). IFA uses two McAbs against the PEDV and TGEV, respectively. PEDV-specific immunofluorescence was detected in PEDV-infected cells by anti-PEDV McAb-1 and anti-PEDV McAb-2 (Figure 2C). TGEV-specific immunofluorescence was detected in TGEV-infected ST cells by anti-TGEV McAb-1 and anti-TGEV McAb-2 (Figure 2D). In contrast, in the mock cells, there was no immunofluorescence staining.

### 3.2 Development of a GICA strip for simultaneous detection of PEDV and TGEV

To develop a rapid and efficient diagnostic test for PEDV and TGEV simultaneously, a novel colloidal gold test strip was generated using two anti-PEDV paired McAbs and two anti-TGEV paired McAbs. In the test strip, anti-PEDV McAb-1 and anti-TGEV McAb-1 as detectors were biotinylated and labeled with colloid gold, then applied to the conjugate pad. Anti-PEDV McAb-2 and anti-TGEV McAb-2 were dispensed on the nitrocellulose membrane to capture the PEDV and TGEV antigen. When the sample was added to the sample pad, PEDV and TGEV antigens migrated into the conjugate pad and were captured by the colloid gold-labeled McAb-1. The binding of streptavidin from the sample pad at a ratio of 1:4 to the biotin coupled with anti-PEDV McAb-2 and anti-TGEV McAb-2 amplified the colloidal gold signal. The complex on the nitrocellulose membrane, captured by anti-PEDV McAb-2 and anti-TGEV McAb-2, generated the T1 and T2 lines and the excess colloidal gold compound captured by the anti-mouse IgG antibody generated the control line C (Figure 1C).

### 3.3 Specificity of the GICA strip

**Figure 3:**
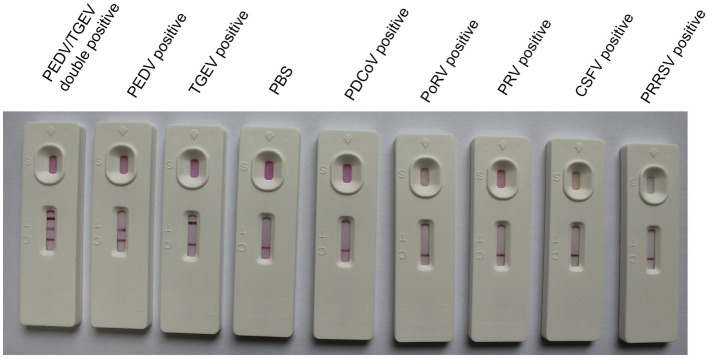
Specificity of the GICA strip.

To identify the specificity of the IC test strip, the test strips were used to detect standard negative and positive samples, and samples containing PDCoV, PoRV, PRV, CSFV, and PRRSV. If the sample contained PEDV or TGEV, the T1 or T2 line and the C line became visible. For other samples that did not contain PEDV or TGEV, only a single-colored control line was visible (Figure 3), indicating that this method had high specificity for detecting PEDV and TGEV. There was no cross-reaction in the detection of other viruses.

### 3.4 Sensitivity of the GICA strip

**Figure 4:**
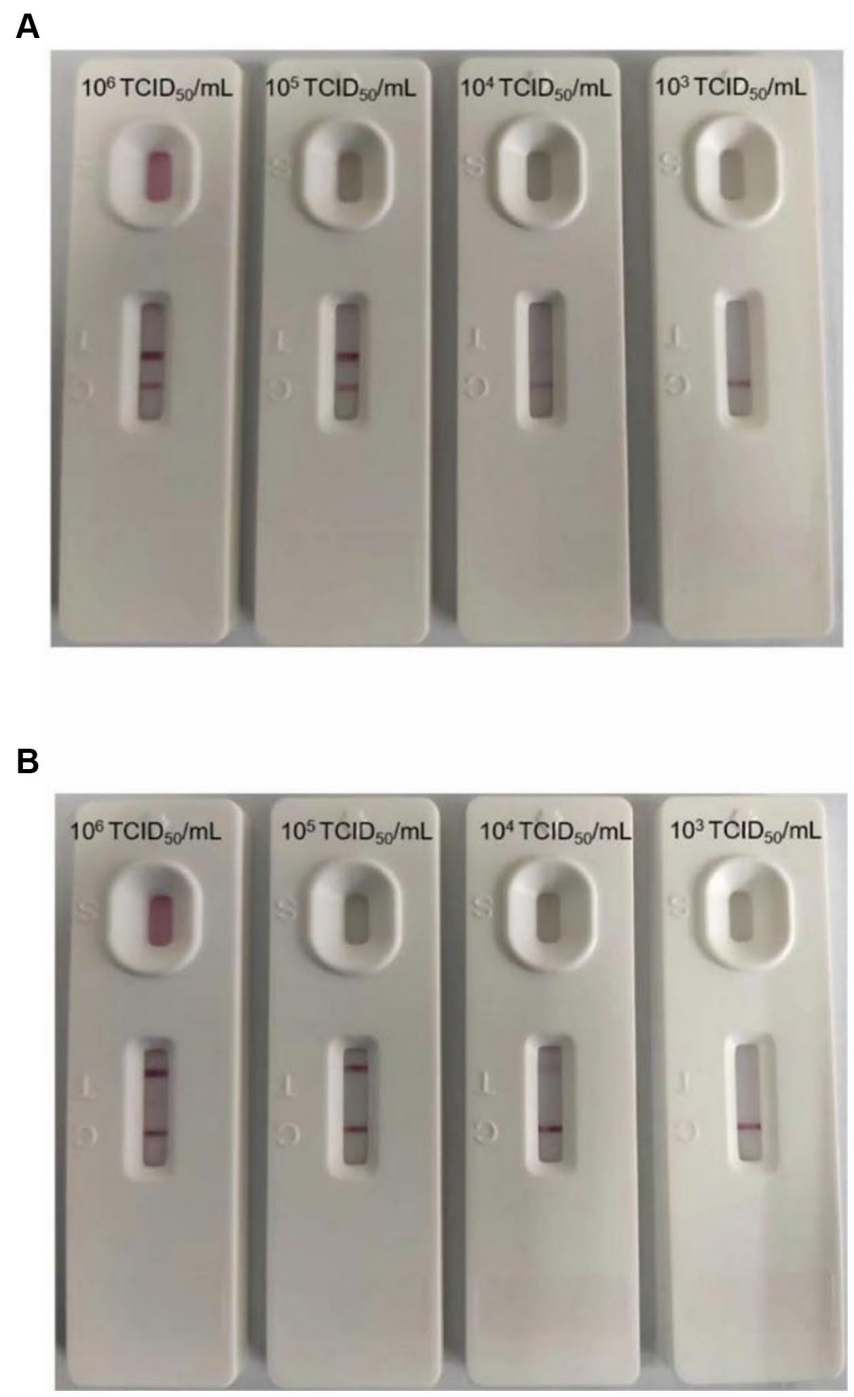
Minimum detection limit of the GICA strip. **(A)** The dilution range of the PEDV was 10^6^–10^3^ TCID_50_/mL, and the lowest detection line was 10^4^ TCID_50_/mL. **(B)** The dilution range of the TGEV was 10^6^–10^3^ TCID_50_/mL, and the lowest detection line was 10^4^ TCID_50_/mL.

The sensitivity of the test strip was analyzed by testing serially diluted cell cultures of PEDV and TGEV (PEDV and TGEV were serially diluted to 1.0 × 10^6^, 1.0 × 10^5^, 1.0 × 10^4^, and 1.0 × 10^3^ TCID_50_/mL). The prepared strips were used to test different concentrations of PEDV and TGEV. The results showed that the minimum virus detectable by the test strips was approximately 1.0 × 10^4^ TCID_50_/mL for PEDV and 1.0 × 10^4^ TCID_50_/mL for TGEV, respectively (Figures 4A,B). Similar results were observed in three repetitions. These results showed that the test strip had high sensitivity for detecting PEDV and TGEV.

### 3.5 Stability of the GICA strip

**Figure 5:**
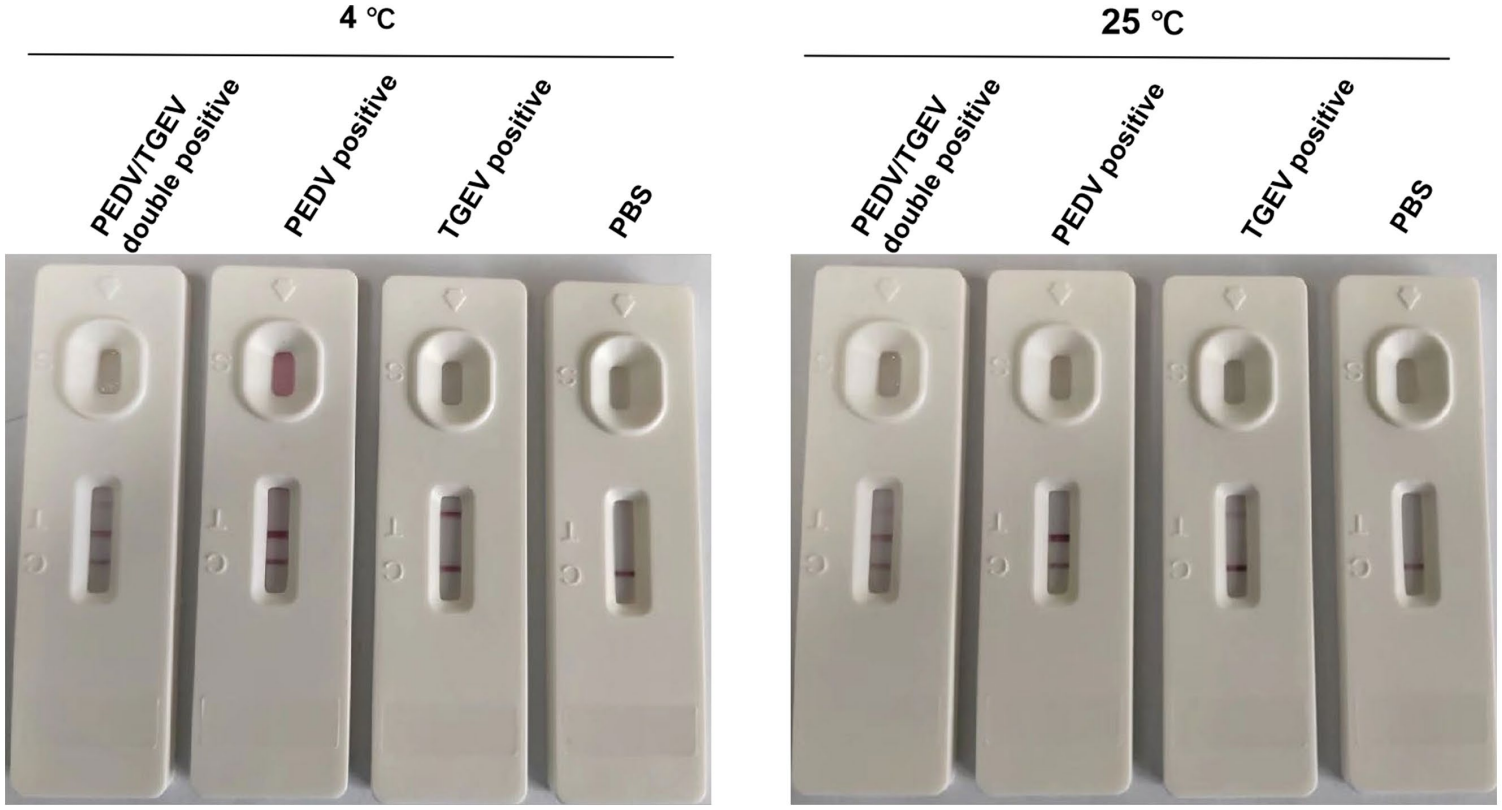
Stability of the GICA strip. The kit was stable at 4 or 25°C for 1 year.

To evaluate the stability of the test strip, prepared strips from the same batch were stored at 4°C or 25°C for 12 months, following which cell cultures of PEDV and TGEV were detected. The results showed that the strips were stable for 1 year at 4°C or 25°C, indicating that they are suitable for practical applications (Figure 5).

### 3.6 Applicability of the GICA strip for clinical samples

To evaluate the applicability of the colloidal gold test strip for clinical samples, a total of 121 clinical samples were detected using these strips. The strips successfully detected positivity for PEDV, TGEV, and double positives in 26.45% (32/121), 4.95% (6/121), and 3.31% (4/121) of samples, respectively. Simultaneously, all clinical samples were assessed using RT-PCR, which detected positivity for PEDV, TGEV, and double positives in 49.58% (60/121), 11.57% (14/121), and 6.62% (8/121) of samples, respectively (Table 3). These data demonstrated that the colloidal gold test strip can be used efficiently for the simultaneous detection of PEDV and TGEV in clinical samples.

**Table 3:** Test results of clinical samples.

Detection method	PEDV		TGEV		PEDV-TGEV	
	GICA	RT- PCR	GICA	RT- PCR	GICA	RT- PCR
No. of positive samples	52	60	12	14	7	8
No. of negative samples	69	61	109	107	114	113
Positive rate	42.9%	49.7%	9.9%	11.6%	5.8%	6.61%
Positive coincidence rate	86.6%		85.7%		87.5%	
Negative coincidence rate	100%		100%		100%	

## 4 Discussion

Although commercial vaccination is available to prevent PEDV and TGEV, pig diarrhea is responsible for considerable economic losses to the swine industry, especially affecting piglets (Katsuda et al., 2006). Diarrhea in piglets is mainly caused by coronaviruses, especially TGEV and PEDV (Zhang et al., 2019; Liu and Wang, 2021). Moreover, in recent years, mixed infection by PEDV and TGEV has become common (Zhao et al., 2014; Huang et al., 2019). At present, prevention and early detection remain effective ways to control this infectious disease, and many routine detection methods for PEDV and TGEV have been developed (Diel et al., 2016). However, no rapid IC strip for detecting PEDV and TGEV simultaneously has been reported.

GICA is a rapid and simple method to detect pathogens in the field, and it has been widely used to detect many pathogens, such as PEDV (Lyoo et al., 2017), African swine fever virus (Zhang et al., 2021), H7N9 influenza viruses (Sun et al., 2018), and severe acute respiratory distress syndrome coronavirus 2 (Oshiro et al., 2021). The amino acid sequences of PEDV and TGEV N proteins are well conserved (Brian and Baric, 2005; Jung and Saif, 2015); therefore, N proteins are an appropriate target in the development of diagnostic methods for PEDV and TGEV.

In the present study, we developed a GICA based on the lateral flow platform using specific McAbs against PEDV and TGEV N proteins for antigen capture and detection. Our data showed that the strip was efficient compared with RT-PCR detection. The strip was highly specific for detecting viruses in different clinical samples, including stools and intestine contents. Many molecular diagnosis methods have been reported and are used to detect PEDV and TGEV in the laboratory with high sensitivity and accuracy, and they play an important role in the investigation of pathogen molecular epidemiology (Paton et al., 1997; Song et al., 2006; Kim et al., 2007). Compared with these molecular diagnosis methods, GICA is more convenient and faster for the detection of diarrheal infectious diseases in pigs in the field. However, RT-PCR targeting RNA and GICA targeting N protein cannot be directly compared because the sensitivity of GICA is lower than that of RT-PCR. This indicates that the GICA is not suitable for epidemiological surveillance, but it is easily handled without any special equipment, which can be a complement for clinical testing. In the following study, more samples from diarrheal pigs will be investigated to validate the GICA for clinical diagnosis on-site.

## 5 Conclusion

In conclusion, we successfully established a GICA kit for simultaneous detection of PEDV and TGEV within 10 min. The kit has the advantages of strong specificity, good stability, and ease of handling without any special equipment, making it a valuable complement for clinical testing.

## Data availability statement

The datasets presented in this study can be found in online repositories. The names of the repository/repositories and accession number(s) can be found in the article/supplementary material.

## Ethics statement

The animal studies were approved by the Jiangsu Academy of Agricultural Sciences Experimental Animal Ethics Committee. The studies were conducted in accordance with the local legislation and institutional requirements. Written informed consent was obtained from the owners for the participation of their animals in this study.

## Author contributions

JiZ: Writing – original draft, Writing – review & editing. WWu: Writing – original draft, Writing – review & editing. DW: Writing – original draft, Writing – review & editing. WWa: Software, Validation, Writing – review & editing. XC: Methodology, Investigation, Writing – review & editing. YL: Methodology, Investigation, Writing – review & editing. JL: Data curation, Formal analysis, Writing – review & editing. BF: Data curation, Formal analysis, Writing – review & editing. JuZ: Conceptualization, Visualization, Writing – review & editing. RG: Methodology, Investigation, Writing – review & editing. XZ: Conceptualization, Visualization, Writing – review & editing. BL: Funding acquisition, Project administration, Supervision, Writing – review & editing.
