# Supplementary material for: Development of a colloidal gold immunochromatographic strip for the simultaneous detection of porcine epidemic diarrhea virus and transmissible gastroenteritis virus
Source: Front Microbiol. 2024 Jun 19;15:1418959. doi: 10.3389/fmicb.2024.1418959 (PMC11220158; doi:10.3389/fmicb.2024.1418959)

Supplementary material

Fig. S1 Results of RT-PCR for detecting PEDV

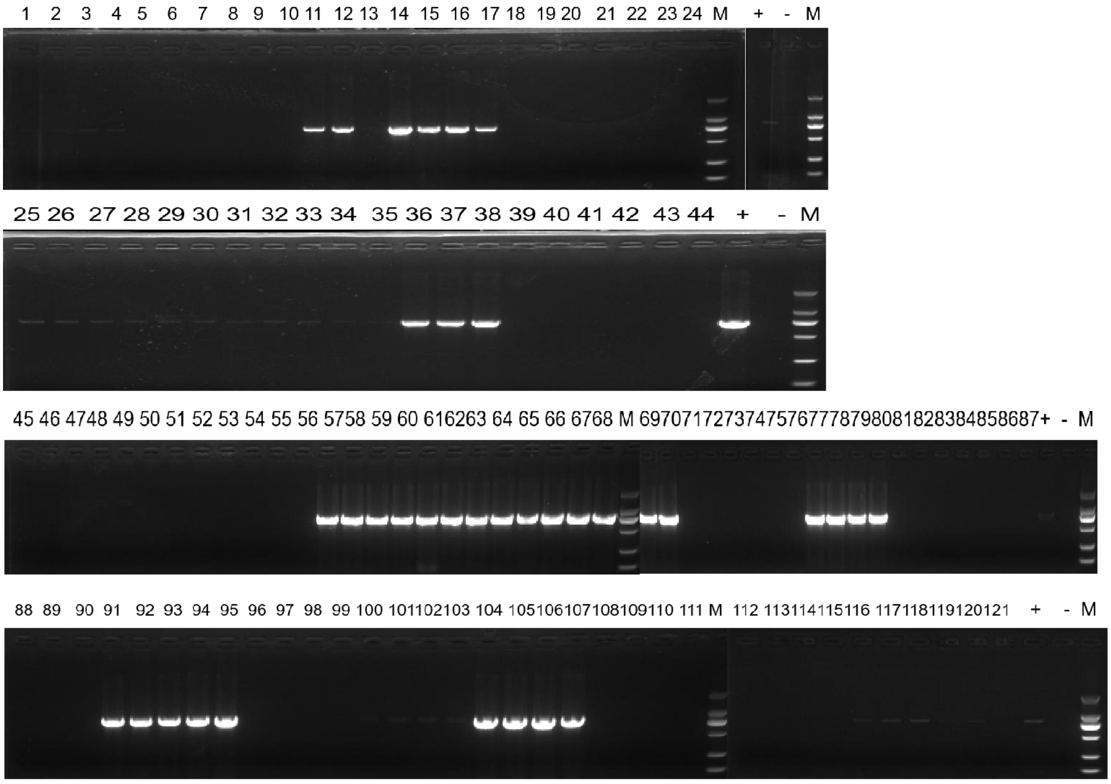

Fig. S2 Results of RT-PCR for detecting TGEV

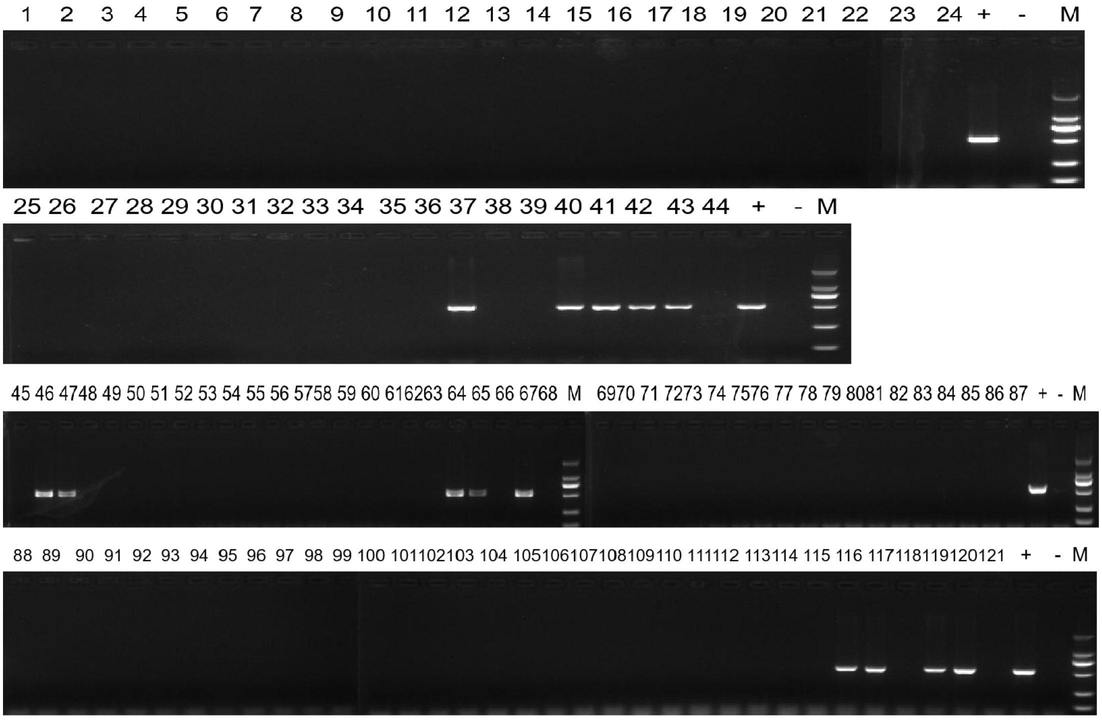

Fig S3 The SDS-PAGE of purified monoclonal antibody.

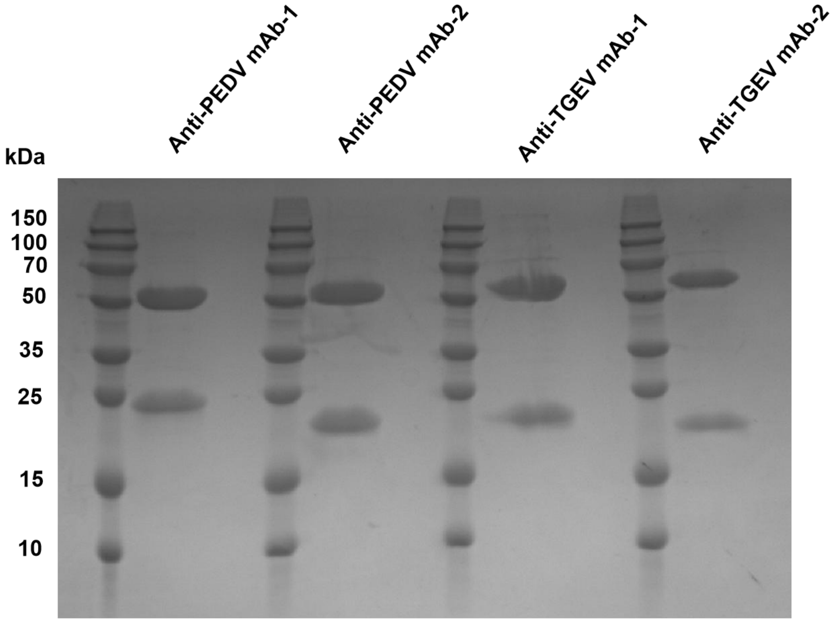

Supplement: Supplementary file 1 [file Image_1.pdf]
